# Sensors for 3D Imaging: Metric Evaluation and Calibration of a CCD/CMOS Time-of-Flight Camera

**DOI:** 10.3390/s91210080

**Published:** 2009-12-11

**Authors:** Filiberto Chiabrando, Roberto Chiabrando, Dario Piatti, Fulvio Rinaudo

**Affiliations:** 1 DINSE, Politecnico di Torino, Viale Mattioli, 39, 10125, Torino, Italy; E-Mail: filiberto.chiabrando@polito.it; 2 DEIAFA, Facoltà di Agraria, Università degli Studi di Torino, Via L. da Vinci, 44, 10095 Grugliasco (TO), Italy; E-Mail: roberto.chiabrando@unito.it; 3 DITAG, Politecnico di Torino, C.so Duca degli Abruzzi, 24, 10129, Torino, Italy

**Keywords:** Time-of-Flight camera, range imaging, calibration, systematic errors, CCD/CMOS sensor

## Abstract

3D imaging with Time-of-Flight (ToF) cameras is a promising recent technique which allows 3D point clouds to be acquired at video frame rates. However, the distance measurements of these devices are often affected by some systematic errors which decrease the quality of the acquired data. In order to evaluate these errors, some experimental tests on a CCD/CMOS ToF camera sensor, the SwissRanger (SR)-4000 camera, were performed and reported in this paper. In particular, two main aspects are treated: the calibration of the distance measurements of the SR-4000 camera, which deals with evaluation of the camera warm up time period, the distance measurement error evaluation and a study of the influence on distance measurements of the camera orientation with respect to the observed object; the second aspect concerns the photogrammetric calibration of the amplitude images delivered by the camera using a purpose-built multi-resolution field made of high contrast targets.

## 1. Introduction and State of the Art

In recent years, a new generation of active cameras, based on the Time-of-Flight principle (ToF), has been developed. These devices are usually characterized by no more than a few thousands of tens of pixels, a maximum unambiguous measurement range up to thirty meters and small dimensions. The main advantages with respect to other 3D measurement techniques are the possibility to acquire data at video frame rates and to obtain 3D point clouds without scanning and from just one point of view.

ToF cameras usually deliver a range image and an amplitude image with infrared modulation intensities at video frame rates: the range image (or depth image) contains for each pixel the radial measured distance between the considered pixel and its projection on the observed object, while the amplitude image contains for each pixel the strength of the reflected signal by the object. In some cases an intensity image is also delivered, which represents the mean of the total light incident on the sensor (reflected modulated signal and background light of the observed scene). As shown in many previous works [1-7], the distance measurements of ToF cameras are influenced by some systematic errors. In order to model these errors, previous ToF camera calibration approaches performed a linear mapping, adjusting range measurement errors using look-up-tables [3] or splines [4]: in both cases they determined the distance errors using high precision measurement racks. Instead, this work presents a first attempt to obtain with a low-cost custom-made system a distance error model of a ToF camera (SwissRanger-4000 camera) that would be unique for all the SR-4000 camera pixels and which could be applied to data acquired with the standard software supplied with this camera. It is worth noting that all the tests reported in this paper were performed under indoor controlled conditions.

In the following section the ToF measurement principle of range imaging cameras is briefly described. Then, some experimental tests for the camera distance measurement calibration are reported in Section 3. Section 4 reports about the photogrammetric calibration of the amplitude images delivered by the camera using a purpose-built multi-resolution field made of high contrast targets. Finally, some conclusions and future works are reported in Section 5.

## 2. ToF Measurement Principle

Two main variations of the ToF principle have been implemented above all: one measures distance by means of direct measurement of the runtime of a travelled light pulse using arrays of single-photon avalanche diodes (SPADs) [8,9]; the other method uses amplitude modulated light and obtains distance information by measuring the phase difference between a reference signal and the reflected signal [10,11]. While complex readout schemes and low frame rates have prevented the use of SPAD arrays in commercial 3D-imaging products up to now, the second category has already been implemented successfully in several commercially available 3D camera systems. More information about pixel structures and performance limitations of these sensors can be found for instance in [12].

The ToF camera that was tested in this work is a phase shift measurement device. The SR-4000 camera modulates its illumination LEDs (Light Emitting Diodes) at a few tens of MHz and its CCD/CMOS imaging sensor measures the phase of the returned modulated signal at each pixel. The distance at each pixel is determined as a fraction of the one full cycle of the modulated signal, where the distance corresponding to one full cycle (the so called “non-ambiguous range”) is given by:

(1) $$D=\frac{c}{2f_{\mathrm{mod}}}$$

where c is the speed of light and f_mod_ is the modulation frequency of the emitted signal. All the tests described in the following sections were performed using a modulation frequency of 30 MHz (standard setting): this results in a non-ambiguity range of 5.00 m. Different modulation frequencies can be adopted with this camera, with a maximum non-ambiguity range of 10.00 m [13]. A higher modulation frequency might provide better measurement accuracy but a shorter non-ambiguous range. Therefore, as a general rule, it can be stated that the modulation frequency should be adjusted based on the maximum target distance, remembering that reflective objects beyond the non-ambiguous range are frequently aliased [14].

**Figure 1.**
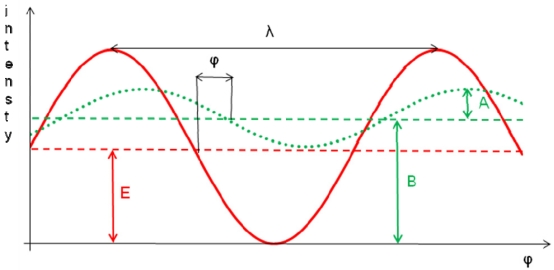
Scheme of the phase shift measurement principle (the solid curve represents the emitted signal while the dotted one represents the received signal).

A scheme of the phase shift measurement principle is reported in Figure 1: E is the amplitude of the emitted modulated signal, B is the mean intensity of the received signal and A is its amplitude. The received signal is offset-shifted in intensity with respect to the emitted signal mainly because of additional background light [15].

The received signal is sampled four times in each cycle, at ¼ period phase shifts, *i.e.*, 90° phase angle. From the four samples (A0, A1, A2, A3), the parameters A and B and the phase φ can be calculated:

(2) $$A=\frac{\sqrt{{\left(A0-A2\right)}^{2}+{\left(A1-A3\right)}^{2}}}{2}$$

(3) $$B=\frac{A0+A1+A2+A3}{4}$$

(4) $$\varphi =\arctan\left(\frac{A0-A2}{A1-A3}\right)$$

The distance R is then derived from the phase φ:

(5) $$R=\frac{c}{4\pi \cdot f_{\mathrm{mod}}}\cdot \varphi$$

The amplitude A may be used as a measure of quality of the distance measurement [10], or simply to generate a grayscale image of the observed scene.

In order to give an idea of the characteristics of the camera tested in this work, the principal specifications of the SR-4000 camera are reported in Table 1. For more details about camera specifications see [13].

## 3. Distance Calibration

Some experimental tests relative to the calibration of the distance measurements delivered by the SR-4000 camera are reported in the following sub-sections. In our tests all the data were acquired with the SR_3D_View software provided with the camera, which delivers point coordinates (X, Y and Z), amplitude data of the object that has to be detected and a confidence map of the distance measurements. In particular, the confidence map is obtained using a combination of distance and amplitude measurements and their temporal variations: it represents a measure of probability or ‘confidence’ that the distance measurement for each pixel is correct, so it can be useful to select regions containing high quality measurements or to reject low quality ones.

### 3.1. Warm up Time Period Evaluation

ToF camera measurements are influenced by the internal temperature of the measurement system [3,7,16]. In order to determine the camera warm up time period necessary to achieve distance measurement stability, the following test was carried out.

The SR-4000 camera was set up on a photographic tripod, with the front of the camera parallel to a white wall. After turning on the camera, five consecutive frames were acquired every five minutes for two hours of camera working. The test was carried out at several distances (and integration times) between the front of the camera and the wall. The integration time is the length of time that the pixels are allowed to collect light. Data were acquired using an integration time equal to the “auto integration time” suggested by the SR_3D_View software. This software allows one to automatically adjust the integration time depending on the maximum amplitudes present in the current image. This setting was used in order to avoid pixel saturation and to achieve a good balance between noise and high frame rate.

**Figure 2.**
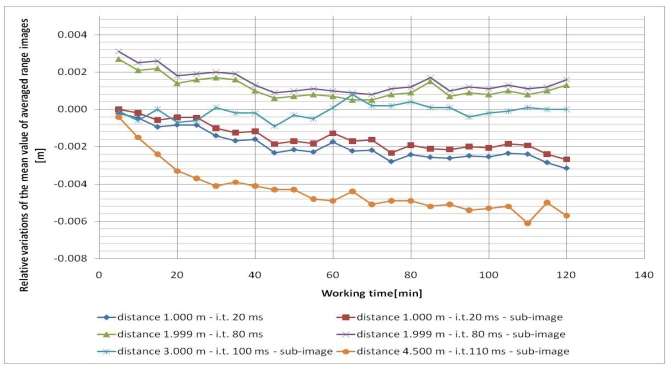
Relative variations of the mean value of averaged range images during the working time of several tests (i.t. means integration time).

**Figure 3.**
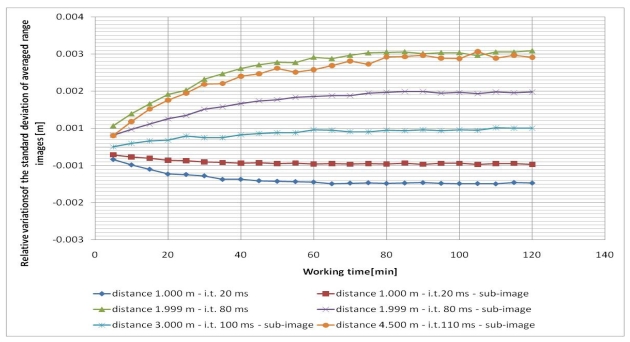
Relative variations of the standard deviation of averaged range images during the working time of several tests (i.t. means integration time).

In all cases, the five frames (range images) acquired each time were averaged pixel by pixel in order to reduce the measurement noise. Since the camera was not moved from its position in each test, variations during the working time of the mean and standard deviation of the averaged range images were considered. Since the tests were performed at different distances (and integration times), the relative variations of the mean and standard deviation, with respect to their initial values, were considered for each test in order to compare them (Figure 2 and Figure 3). In all cases a central sub-image of 84 × 96 pixels was considered, while in two cases (when the wall filled the entire range image), also the entire image of 176 × 144 pixels was considered.

As can be observed from Figures 2 and 3, both the mean value and the standard deviation of the distance measurements vary during working time: a maximum variation of about -6 mm was detected for the mean value, while a maximum variation of about 3 mm was measured for the standard deviation. Since the calculated variations are nearly constant after forty minutes of camera working, one can stand that a warm up period of forty minutes can be sufficient to achieve a good measurement stability of the SR-4000 camera.

### 3.2. Systematic Distance Measurement Error Evaluation

**Figure 4.**
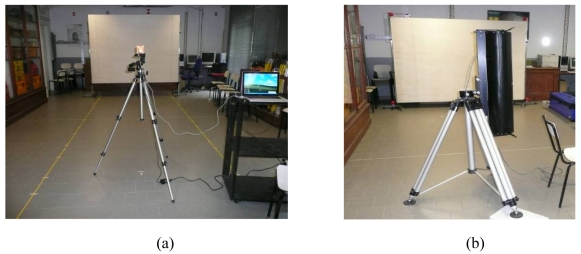
(a) Data acquisition with the SR-4000 camera. (b) Laser scanner survey of the panel with Mensi S10.

In order to evaluate the systematic distance measurement errors of the SR-4000 camera, a custom-made system was set up. The camera was positioned parallel to a vertical plywood panel (1.85 m × 2.52 m) supported by two adjustable tripods. The distance between the camera front and the panel was accurately measured using two parallel metal tape-measures (Figure 4a). This solution was designed in order to obtain an economic way to estimate the camera distance measurement accuracy. Unfortunately, since the purchased panel wasn't perfectly flat, laser scanner and total station surveys of the plywood were performed in order to create a detailed model of the panel. A Mensi S10 laser scanner, which acquired about 780,000 points with sub-millimetric precision, was employed (Figure 4b).

After the camera warm up, the panel was positioned each five centimeters in the 0.50 ÷ 4.50 m distance range between the camera front and the plywood. Thirty consecutive frames were acquired for each panel position, using an integration time equal to the “auto integration time” suggested by the SR_3D_View software.

The acquired data were processed using a custom-made Matlab^®^ application in order to evaluate the distance measurement errors and estimate a first distance error model. Since the panel did not fill the entire images delivered by the camera in almost half of the test distance gap, we limited our analysis to 7,921 pixels which are contained in a sub-image of 89 × 89 pixels centered with respect to the central pixel of the camera sensor. For pixel in row *i* and column *j* position, let us define the following terms:

(6) $$h_{i,j}=\frac{\overset{n}{\underset{f=1}{\text{∑}}}m_{i,j,f}}{n}-r_{i,j}$$

(7) $$g=\frac{\overset{89}{\underset{i=1}{\text{∑}}}\overset{89}{\underset{j=1}{\text{∑}}}h_{i,j}}{7921}$$

(8) $$s=\frac{\overset{89}{\underset{i=1}{\text{∑}}}\overset{89}{\underset{j=1}{\text{∑}}}\overset{30}{\underset{f=1}{\text{∑}}}r_{i,j,f}}{7921}$$

where *h_i,j_* is the discrepancy for pixel *i,j,f* represents a generic frame, *n* = 30 is the number of acquired frames, *m_i,j,f_* is the measured distance for pixel *i,j* at the *f*th frame and *r_i,j_* is the real distance between pixel *i,j* and its orthogonal projection on the panel. This latter term was obtained from accurate metal tape-measures measurements combined with the panel model in order to take into account the panel curvatures.

**Figure 5.**
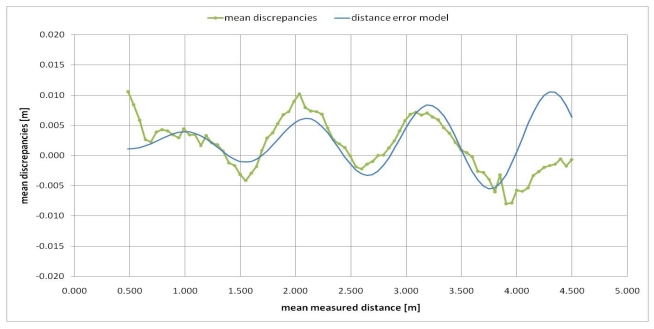
Variation of the mean values of the discrepancies (*g*) of all the considered pixels according to the mean measured distance (*s*) and distance error model.

The variation of the mean values of the discrepancies (*g*) of all the considered pixels according to the mean measured distance (*s*) is reported in Figure 5. As can be observed from this figure the discrepancies between measured distance and real distance show a maximum value of 11 mm and a minimum value of −8 mm. These measurement errors are smaller than those of other Swiss Ranger cameras tested in previous works [3,7,17,18]. Besides, some tests similar to the ones reported in [17,18] were performed, which highlighted that the SR-4000 camera measurements are not affected by the scattering artifacts caused by multiple internal reflections occurring inside the SR-3000 line of sensor, which significantly limited its distance measurement accuracy [17,18].

However, from Figure 5 one can observe that a systematic trend of the measurement errors still remains which needs to be corrected. We modeled these measurement errors (discrepancies) with the following distance error model (e):

(9) $$e=\lambda_{0}+\lambda_{1}\cdot m\cdot \sin(\lambda_{2}\cdot m+\lambda_{3})$$

where *m* is the pixel measured distance, λ_0_ is a constant error and λ_1_ represents a scale factor which multiplies a “wiggling error” modeled by a sinusoidal function (λ_2_ = angular frequency, λ_3_ = phase shift).

### 3.3. Control Measurements

**Figure 6.**
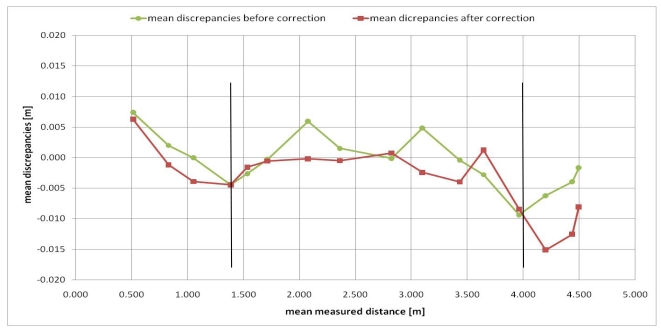
Mean values of the discrepancies (*g*) of all the considered pixels before and after distance error model correction.

In order to obtain a first check of the proposed distance error model, a procedure similar to the one adopted in Section 3.2 was adopted. After the camera warm up, the panel was randomly positioned at several distances from the camera. After averaging the thirty frames acquired for each position, each pixel distance measurement was corrected with the distance error model (9). The mean values of the residual discrepancies for 7,921 pixels are represented in Figure 6.

Figure 6 shows that, after applying the proposed distance error model (9), the absolute values of residual discrepancies are smaller than the discrepancies without correction in the 1.5–4.0 m distance measurement range. For measurement distances up to 1.5 m and longer than 4.0 m, our distance error model generates greater absolute residual discrepancy values; in fact, as can be observed from Figure 5, this model badly fits distance deviations especially for longer distances than 4.0 m.

Since the 1.5–4.0 m distance measurement range is the most useful measurement range for our purposes (architectural and archaeological surveys, object modeling and 3D indoor scene reconstruction), the proposed model is suitable for our applications. Nevertheless, we will try to improve this aspect in future works. For instance, the measurement errors for distances shorter than 1.5 m and longer than 4.0 m could be modeled by polynomial functions.

### 3.4. Influence of Camera Orientation on Distance Measurements

**Figure 7.**
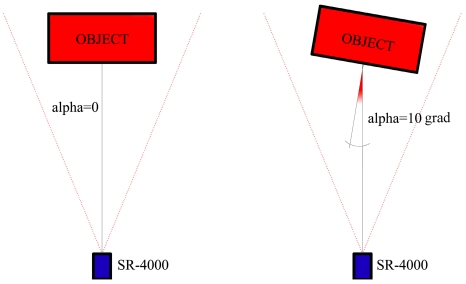
Alpha angle between camera optical axis and the normal to the object surface.

The signal emitted by the camera impinges the observed object with an angle which depends on the camera orientation with respect to the normal of the object surface. We can define alpha as the angle between the camera optical axis and the normal to the object surface, as shown in Figure 7.

Some previous works have already examined the influence of the emitted signal angle of incidence on distance measurement precision [1-19]. While in [19] only some statements are given about the influence of the angle of incidence on the residuals obtained after an automated segmentation of range videos acquired under simplified real-world conditions, in [1] a more significant test is performed. In this latter work, range images were acquired with different constant integration times of a reference panel which was rotated; in this way, the distance measurement precision decreased for an increasing angle because there was less reflected light with respect to the frontal position of the panel. Instead, in this work, we analyze this aspect from a more practical point of view: our analysis deals with data acquired with the SR_3D_View software using the “auto integration time”, so changing the integration time for each object position as a generic user could do acquiring data of the object to be surveyed.

**Figure 8.**
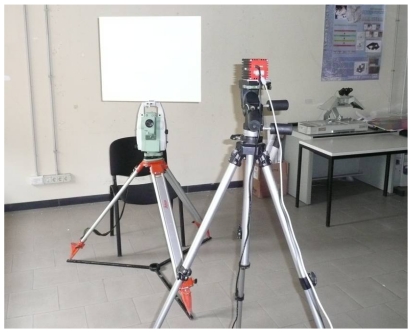
System used to evaluate the influence of the alpha angle on camera distance measurements.

In order to evaluate if there is an influence of the alpha angle on the precision of distance measurements acquired in this way, the following system was set up. The camera was positioned on a photographic tripod, with the camera front parallel to a Plexiglas panel, which was fixed to a Leica TS (Figure 8); the panel was covered with white sheet.

**Figure 9.**
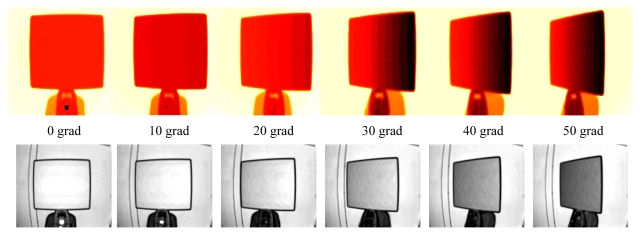
Range images (top) and amplitude images (bottom) acquired with different alpha angles in clockwise direction (arbitrary color scales).

After the camera warm up, using the Leica TS, the panel was accurately rotated each two grad in the 0 ÷ 50 grad rotation interval (Figure 9), both in clockwise direction and counterclockwise direction, while the SR-4000 camera was fixed. Fifty consecutive frames were acquired for each panel position, using an integration time equal to the “auto integration time” suggested by the SR_3D_View software. The distance between the panel and the camera was about 1.6 m.

In order to accurately estimate the distribution of the distance measurements around their mean value, a reference plane for each panel position was estimated after outlier elimination from the acquired range images thanks to a robust estimator, the Least Median Squares (LMS) estimator [20]. This estimator has a high breakdown point, which means that it can discriminate outliers and leverage points up to a percentage of 50% of the considered data. The parameter which has more influence on the LMS results is the threshold value of rejection L, that represents a preliminary hypothesis on the percentage of outlier contamination. After testing this estimator on several randomly generated range images containing different percentages of outliers, we adopted a threshold value of rejection L = 1.5.

**Figure 10.**
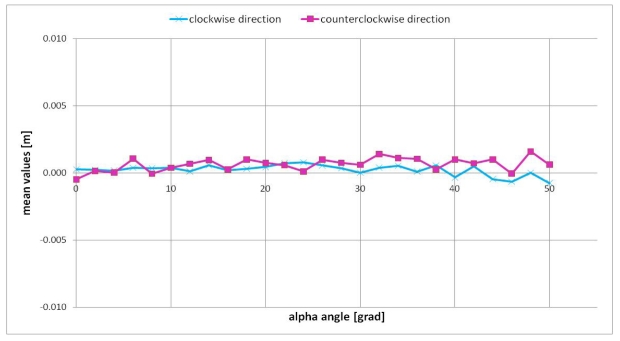
Mean values of the differences between range image and estimated reference plane.

**Figure 11.**
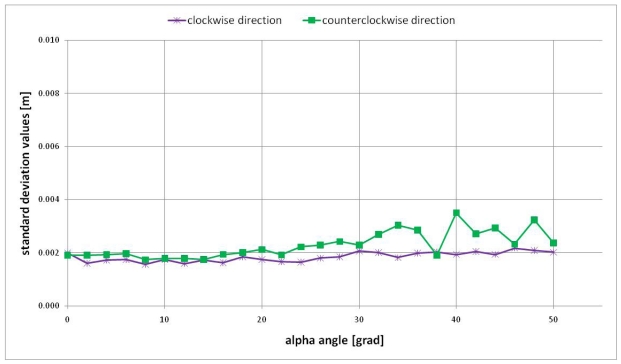
Standard deviation values of the differences between range image and estimated reference plane.

The LMS estimator was applied on a sub-image of 65 × 61 pixel dimensions, which was centered with respect to the panel centre in each position. Thanks to this estimator it was possible to select some reliable points into the sub-image which were necessary for a robust plane estimation. Then the differences between the range image (obtained after averaging fifty frames) and the estimated reference plane were calculated for each panel position, always considering the sub-image of 65 × 61 pixel dimensions. The mean and standard deviation values of that differences are reported in Figures 10 and 11 respectively. In the case of alpha angles larger than fifty grad the area of the panel was too small for a reliable estimation of a reference plane, so our analysis was limited to fifty grad in both directions.

From Figure 10 one can observe that the mean value of the differences between the estimated plane and the SR-4000 distance measurements shows small fluctuations around the zero value according to the alpha angle: these small fluctuations are limited to about 2 mm in both clockwise and counterclockwise directions. Instead, the standard deviation value varies according to the alpha angle (Figure 11): this variation is contained in about 2 mm. This trend is justified by the adopted procedure: since the data were acquired with the “auto integration time” for each panel position, the reduction of the amount of reflected light from the panel is limited to about 20% with respect to the reflected light from the initial position (alpha angle equal to zero) (Figure 9 bottom). The distance measurement standard deviation is in inverse proportion with respect to the amplitude of the reflected light [15,16,21]; therefore, an amplitude reduction of about 20% will approximately result in an increment of about 25% of the distance measurement standard deviation. Since the typical standard deviation value of the distance measurements is 4 mm [13], a 25% increment of that value is negligible. This aspect is confirmed by the afore reported results.

In conclusion, adopting the “auto integration time” for data acquisition, there is no appreciable variation of the distance measurement precision for camera orientations included within the considered alpha angle interval.

## 4. Photogrammetric Calibration

**Figure 12.**
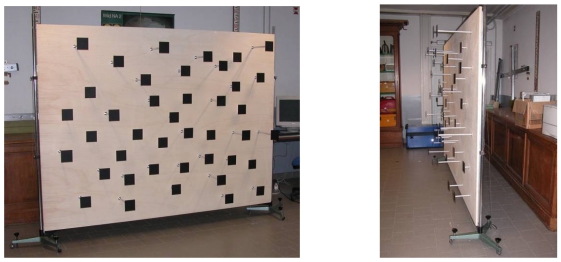
Multi-resolution field made of forty-one high contrast targets with different depths.

The photogrammetric calibration was considered separately from the distance measurement calibration, according to some previous works [3,4,22]. In [3], a planar test-field whose targets are represented by Near-Infra-Red (NIR) LEDs was built, while in [4-22] a planar test-field with a black and white checkerboard pattern was employed. In order to have targets well distributed in the three dimensions, a purpose-built multi-resolution field made of forty-one high contrast targets with different depths was created in our case (Figure 12). All the target coordinates were carefully measured with a total station survey in order to obtain accurate control point coordinates.

For the photogrammetric calibration the iWitness^©^ software was employed, which uses the ten-parameter “physical” model commonly employed in digital close-range photogrammetry [23]. The interior orientation parameters are principal distance (c), principal point offsets (x_0_, y_0_), three coefficients of radial distortion (K_1_, K_2_, and K_3_), two decentering distortion coefficients (P_1_, P_2_) and the affinity non-orthogonality parameters (b_1_, b_2_, rarely employed in CCD cameras). The following expression is employed for the estimation of the radial distortion correction (*dr*):

(10) $$dr=K_{1}\cdot r^{3}+K_{2}\cdot r^{5}+K_{3}\cdot r^{7}$$

where r is the radial distance between the principal point and a generic point of the image. The K_i_ coefficients are usually highly correlated, with most of the error signal generally being accounted for by the cubic term K_1_ r^3^. The K_2_ and K_3_ terms are typically included for photogrammetric and wide-angle lenses, and in higher-accuracy vision in metrology applications [24].

In our case the following parameters were estimated: principal distance (c), principal point coordinates (x_0_, y_0_), radial distortion coefficients (K_1_, K_2_, K_3_) and decentering distortion coefficients (P_1_, P_2_).

Different sets of amplitude images of the multi-resolution field were acquired, according to the typical conventions which should be followed in the case of camera self-calibration: the network should display moderate to large convergence angles between different images with a sufficient number of points imaged in more than two photographs; moreover, the images should be acquired with different orientations within the network (in both 90° ‘portrait’ and ‘landscape’ orientations) and the object feature points should be well distributed in three dimensions.

In this work three different amplitude image sets were acquired in order to obtain reliable results. The comparison of the obtained results confirmed the stability of the camera internal parameters, which are reported in Table 2.

**Figure 13.**
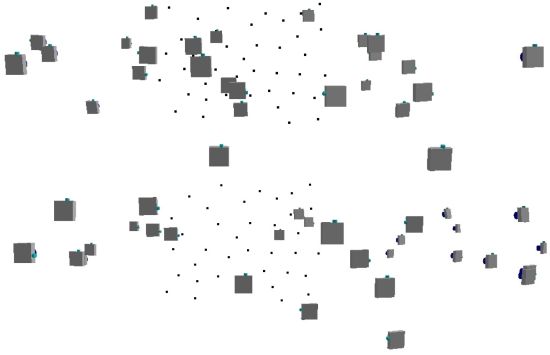
Recovered camera positions for two of the three amplitude image sets employed for the camera photogrammetric calibration.

The final standard deviations of the computed control point coordinates using the calibration parameters are σ_x_ = 0.7 mm, σ_y_ = 0.6 mm and σ_z_ = 1.5 mm. The recovered camera positions for two of the three amplitude image sets employed during the calibration procedure are displayed in Figure 13.

**Figure 14.**
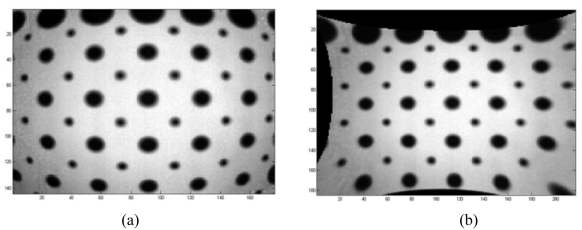
(a) Amplitude image of a test field obtained after averaging thirty frames (b) Amplitude image obtained after averaging thirty frames and after correction from lens distortion.

In this case the photogrammetric calibration was only used for lens distortion correction of the amplitude images acquired with the SR_3D_View software. An example of the results obtained with a custom made Matlab^®^ application using the estimated interior orientation parameters on an amplitude image of a test field after averaging thirty frames is reported in Figure 14.

## 5. Conclusions and Future Works

ToF cameras represent an interesting powerful recent tool for many applications which need 3D point clouds, such as mobile-mapping, metric surveys, object modeling, reverse engineering, robot navigation and real time applications [21,25-28].

The 3D imaging technique allows one to generate point clouds such as in the case of the LiDAR technique and photogrammetry but with the great advantage of real time acquisition, low cost and handiness. However, the accuracy and the application fields of ToF cameras are still not comparable with those of the aforementioned techniques and the procedures to achieve suitable metric products with this methodology are still under study.

Previous works and our tests demonstrated that ToF camera measurements are affected by systematic errors which are caused by both their internal components and the observed scene. These errors cannot be fully eliminated, but they can be reduced to some extent thanks to calibration procedures.

For these reasons the main objectives of the present work were the validation and calibration of data acquired using the ToF SR-4000 camera. Two main aspects were separately considered: the calibration of the distance measurements and the photogrammetric calibration of the amplitude images delivered by the camera. Concerning the distance measurement calibration, a camera warm up period of forty minutes was determined in order to achieve distance measurement stability; then, we proposed a distance error model which provides an excellent reduction of distance errors in the 1.5–4.0 m distance measurement range, which is the most useful measurement range for our purposes (architectural and archaeological surveys, object modeling and 3D indoor scene reconstruction). Nevertheless, in future works, we will try to improve the proposed distance error model to make it suitable for the entire camera operating range. For instance, the measurement errors for distances shorter than 1.5 m and longer than 4.0 m could be modeled by polynomial functions.

Moreover, we demonstrated that there is a negligible variation of the distance measurement precision varying the camera orientation in the considered interval of horizontal angles using the “auto integration time” suggested by the SR_3D_View software.

In order to obtain a reliable camera photogrammetric calibration using the amplitude images delivered by the camera, a purpose-built multi-resolution field made of several high contrast targets with different depths was employed and the typical conventions of digital close-range photogrammetry were adopted for image acquisition. The comparison of the obtained results using different sets of amplitude images demonstrated the stability of the estimated camera internal parameters.

In future works, we will perform some tests on the camera response to different object reflectivity in order to eventually integrate the proposed model with this aspect. Furthermore, some tests have been already performed for the ToF camera application to real object surveys and for the integration of the acquired data with the radiometric content obtained from digital images achieved with external devices.

## Figures and Tables

**Table 1.** SR-4000 specifications.

**Pixel array size [-]**	176 (h) × 144 (v)	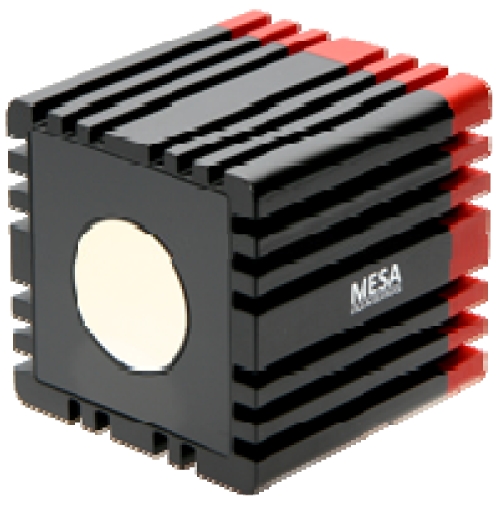
**Field of view [°]**	43.6 (h) × 34.6 (v)	
**Pixel pitch [μm]**	40	
**Illumination wavelength with standard settings [nm]**	850	
**Working range with standard settings [m]**	0.3 ÷ 5.0	
**Maximum frame rate [fps]**	54	
**Dimensions [mm]**	65 × 65 × 68	
**Weight [g]**	470	

**Table 2.** Estimated internal parameters of the SR-4000 camera.

***Principal distance***	c = 9.980 mm
***Principal point offsets***	x_0_ = 0.004 mm
	y_0_ = 0.144 mm
***Radial distortion coefficients***	K_1_ = 1.0780e−002
	K_2_ = −1.0478e−004
	K_3_ = −1.2968e−005
***Decentering distortion coefficients***	P_1_ = −6.1760e−004
	P_2_ = −3.3225e−004
